# QoE Optimization in a Live Cellular Network through RLC Parameter Tuning

**DOI:** 10.3390/s21165619

**Published:** 2021-08-20

**Authors:** Jessica Mendoza, Isabel de-la-Bandera, David Palacios, Raquel Barco

**Affiliations:** 1 Instituto Universitario de Investigación en Telecomunicación (TELMA), Universidad de Málaga, CEI Andalucía TECH E.T.S.I. Telecomunicación, Bulevar Louis Pasteur 35, 29010 Málaga, Spain; ibanderac@ic.uma.es (I.d.-l.-B.); rbarco@uma.es (R.B.); 2 Tupl Spain S.L., Tupl Inc., 29010 Málaga, Spain; david.palacios@tupl.com

**Keywords:** end-to-end (E2E) optimization, quality of experience (QoE), radio link control (RLC)

## Abstract

The mobile communication networks sector has experienced a great evolution during the last few years. The emergence of new services as well as the growth in the number of subscribers have motivated the search for new ways to optimize mobile networks. In this way, the objective pursued by optimization techniques has been evolving, shifting from the traditional optimization of radio parameters to the improvement of the quality perceived by users, known as quality of experience (QoE). In mobile networks, the radio link control (RLC) layer provides a reliable link between both ends of the communication and has a great impact on the QoE. In this paper, the optimization of the QoE for users based on the adjustment of the RLC layer is proposed. For this purpose, two typical services demanded by the users of mobile networks have been selected: the real-time video streaming service and file transfer service. For a broader view of the behavior of the QoE in relation to RLC, optimization tests have been carried out in scenarios with different system bandwidths. In this way, the relationship between the QoE and the optimal configuration of RLC in different network load situations has been analyzed. A proof of concept has been carried out to show the capability of this optimization. To that end, both a cellular network simulator and a live cellular network devised for research purposes have been used.

## 1. Introduction

Over the last few years, the number of subscribers as well as the traffic demand on mobile communications networks have been dramatically increasing. With the arrival of the fifth generation (5G) of mobile communications networks, an increment in the number of services, functionalities, and users is expected [1]. The emergence of new 5G services that require many radio resources and the growth of users’ expectations have motivated the search of new techniques to optimize mobile networks.

Traditionally, mobile network optimization tasks have been focused on the analysis and improvement of certain key performance indicators (KPIs) of the radio access network (RAN), such as the call block rate or the call drop rate [2]. With the constant evolution of the technologies used in mobile networks, optimization techniques have been changing over time, focusing increasingly on the quality perceived by final users from a subjective perspective. To this end, optimization based on certain key quality indicators (KQIs) has been recently proposed [3]. KQIs are metrics defined from the computation of different indicators, such as KPIs [4]. KQIs provide information at the application level about the quality of the services offered to the users following an end-to-end vision. Following this line of thought, in [5] a study of possible improvements in mobility in mobile communications networks is presented with the aim of reducing the interruption time of services. The current trend is focused on the optimization of quality of experience (QoE) metrics. QoE metrics can be directly obtained by conducting tests to users or by using mathematical models that are based on KQIs to approximate the opinion of real users. These techniques reflect the final users’ satisfaction. The authors in [6,7,8,9,10] address the optimization of the QoE perceived by the users of a video streaming service from an application layer perspective. In this way, they use such metrics as the video initial time, the receptor buffer state, or the application level transmission rate to evaluate the QoE. In studies such as [11,12,13], a radio resource scheduler based on the QoE perceived by users is proposed. In [14], the adjustment of physical layer parameters is carried out to optimize the QoE.

Due to the typical interference, noise, and fading of wireless communications, the signal sent by a transmitter will become degraded. A radio link control (RLC) layer provides a reliable link between a transmitter and receiver. This layer performs error detection and correction tasks in order to reduce the impact of the signal degradation. In this way, the RLC layer is responsible for the delay–reliability tradeoff and has a great impact on the QoE.

In the literature, some studies have addressed optimization based on the adjustment of RLC configuration parameters. For example, the authors in [15] presented universal mobile telecommunications system (UMTS) technology. A performance evaluation of the user datagram protocol (UDP) and the transmission control protocol (TCP) in two of the RLC operation modes—AM and unacknowledged mode (UM)—is presented in [16]. Nevertheless, in this work, the authors focus on establishing the advantages of using one mode over another, without going into more detail regarding how to optimize the functioning of each of these RLC operation modes. Furthermore, none of these studies discusses the effect that a misconfiguration of the RLC layer may cause on the QoE. In [17], a preliminary analysis of how the RLC configuration parameters affect the QoE perceived by the users is presented, but this work does not propose a method to optimize it.

In this paper, we propose the optimization of the QoE perceived by the users based on the adjustment of the RLC layer’s configuration parameters. To this end, two types of services that present different characteristics have been selected—real-time video streaming and file transfer—allowing us to more deeply study the different operation modes of the RLC layer. To perform the optimization, a controller based on Taguchi’s method (TM) has been used. Although TM has been proposed in the literature to perform optimization tasks for radio network parameters [18], to the best of our knowledge, this is the first work to use TM for QoE optimization in mobile networks. In this sense, this paper extends the contribution of [17] in which an assessment of the impact of the RLC layer configuration on QoE is presented. The main contributions of this paper are detailed as follows.

- First, unlike in the state of the art methods, where the main objective is to conduct a sensitivity study of how RLC behaves and its different modes of operation, this paper proposes the use of an optimizer based on TM to automatically adjust the configuration parameters of RLC in both operating modes of AM and UM.
- Second, the optimization proposed in this article focuses on the improvement of the QoE perceived by network users. Despite the great impact of the RLC layer on the reliable transmission of data and, therefore, on the QoE perceived by users, the optimization of the QoE based on the adjustment of the RLC configuration parameters has not been addressed in the state of the art approaches. In this paper, the proposed optimization scheme provides the values of the configuration parameters that maximize the QoE perceived by a user for both RLC operating modes of AM and UM. Moreover, the optimization has been carried out for different bandwidth values, establishing a relationship between the RLC configuration parameters, the bandwidth value available in the network, and the obtained QoE.
- Third, in order to evaluate the proposed optimization algorithm, a proof of concept has been carried out. This proof of concept includes a set of tests using two different scenarios. On the one hand, a simulated scenario has been used to analyze the QoE improvements achieved by the automatic RLC layer configuration parameter adjustment. On the other hand, a long-term evolution (LTE) testbed (see Appendix A) has been considered to evaluate the proposed algorithm in a real environment. Although the environments used in the proof of concept correspond to LTE environments, the optimization algorithm proposed in this paper could be used in 5G environments due to the great similarity between the RLC layers of both LTE and 5G technologies [19].

The remainder of this paper is organized as follows. In Section 2, a summary of the RLC layer operation as it is described in the Third Generation Partnership Project (3GPP) is provided. In order to understand the effect caused by a bad configuration of the RLC parameters on the applications that make use of the TCP protocol, as in the case of a file transmission service, it is important to know how the TCP congestion control algorithm works. In this sense, Section 3 describes the main functionalities of the TCP layer, with special emphasis on the study of congestion control. The main characteristics, as well as the metrics used to evaluate the QoE in the different types of services, are defined in Section 4. In Section 5, Taguchi’s method, which is used to perform the optimization of the QoE, is presented. The result of the optimization tasks carried out in the simulator and in the real network are shown in Section 6. Finally, Section 7 presents the conclusions inferred from the results.

## 2. Radio Link Control

This section provides an overview of the operation of the RLC layer-2 sublayer following the 3GPP [20] specifications. In the protocol stack, the RLC layer is located above the medium access control (MAC) layer and below the packet data convergence protocol (PDCP) layer. The operation of the RLC layer is controlled by the radio resource control (RRC) layer and is executed by RLC entities located at both the transmitter and receiver. The main functions of this layer are the segmentation and reassembly of service data units (SDUs) into protocol data units (PDUs) and the detection and correction of errors caused by interference, noise, and fading in the transmission medium—the radio interface.

In order to adapt the RLC layer performance to the requirements of the different services, this layer can be configured in one of the following modes: transparent mode, UM, and AM. Transparent and UM modes are considered unidirectional communication modes, since they do not retransmit lost packets. To perform the communication, both a transmitting and a receiving entity at each communication endpoint are required. While the UM mode peforms the segmentation and reassembly of the RLC SDUs before delivering them to adjacent layers, the transparent mode does not. Transparent mode is used to exchange control messages with the RRC layer, and UM is normally used in real-time services, in which limiting delay takes precedence over achieving good reliability values. Real-time services such as voice transfer make use of this RLC mode. AM mode supports the retransmission of lost packets. In this sense, this mode is considered as bidirectional. Thus, the RLC AM entity at each endpoint has a transmitter and a receiver side. AM also performs the segmentation and reassembly of the packets. AM is used for services that require high reliability in the transmission at the expense of higher delay values. Some examples of services using RLC AM are browsing the web or downloading files.

This paper is focused on the study of the UM and AM modes. Next, the main characteristics of these two modes are presented.

### 2.1. Unacknowledged Mode

**Figure 1:**
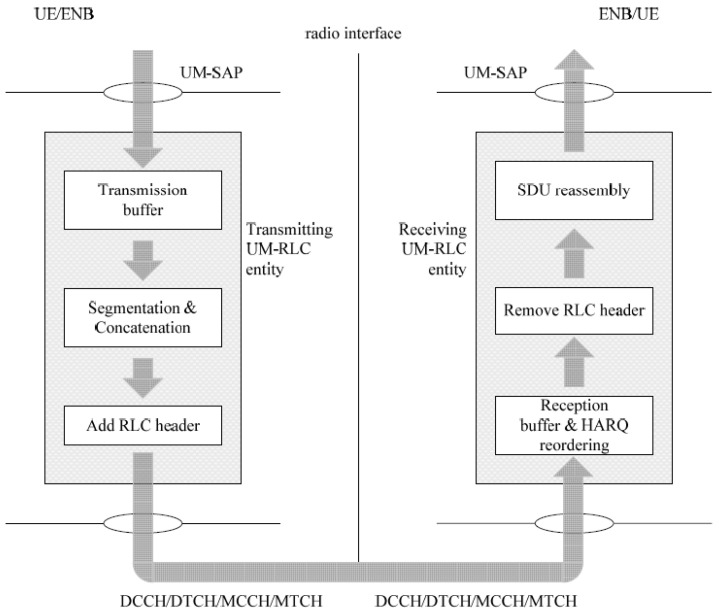
UM RLC entities. Reprinted with permission from ref. [20]. Copyright 2017 3GPP.

As mentioned above, the functionalities of RLC are executed by entities. For the communication to be established, each RLC entity at the transmitter must have a peer at the receiver. When the RLC layer is in the UM operation mode, these entities are called the transmitting UM RLC entity and receiving UM RLC entity, respectively; see Figure 1.

The transmitting UM RLC entity has a transmission buffer in which the RLC SDUs that it receives from the PDCP layer are stored. When the MAC layer observes a transmission opportunity, the transmitting UM RLC entity performs the segmentation and reassembly of the RLC SDUs stored in the transmission buffer, obtaining UM data (UMD) PDUs with an appropriate size to be sent by the MAC layer. Before sending these PDUs, the transmitting UM RLC entity adds an RLC header to the UMD PDUs. The header is used by the receiving UM RLC entity to reorder the UMD PDUs and for error detection tasks.

The receiving UM RLC entity reorders and performs the segmentation and reassembly of the UMD PDU that it receives from the MAC layer; then, it sends the resulting RLC SDUs to the PDCP layer. The UMD PDUs that could not be reordered due to some error detected in the RLC SDUs are discarded. To control how often the reordering and delivery of the packets to the PDCP layer are performed, the receiving RLC UM entity has a timer called t-Reordering. When this timer expires, the receiving RLC UM entity updates its status variables, removes the UMD PDU headers, reassembles the UMD PDUs to obtain RLC SDUs, and delivers them in an orderly manner towards the PDCP layer.

### 2.2. Acknowledged Mode

**Figure 2:**
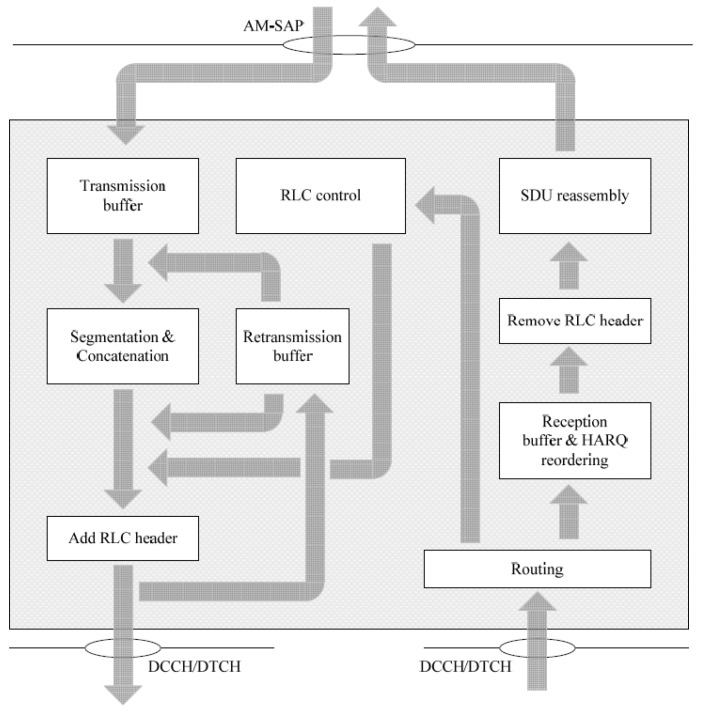
AM RLC entity. Reprinted with permission from ref. [20]. Copyright 2017 3GPP.

Unlike UM RLC entities (a transmitting entity and receiving entity), the AM RLC entities of the transmitter and receiver are the same. Each of these entities will have a transmitting side and a receiving side, as shown in Figure 2.

The transmitting side receives RLC SDUs from the PDCP layer and stores them in a transmission buffer. When the MAC layer observes a transmission opportunity, similarly to the transmitting UM RLC entity, the transmitting side performs the segmentation and reassembly of the RLC SDUs that it has stored, obtaining AM data (AMD) PDUs with an appropriate size to be sent by the MAC layer. Before sending these PDUs to its peer AM RLC entity, the transmitting side adds an RLC header to the AMD PDUs, which are used by the receiver to reorder the AMD PDUs and detect possible errors or missing PDUs. In addition to these functions, the transmitting side supports the retransmission of RLC data PDUs.

The receiving side receives the AMD PDUs from the MAC layer, reorders them, and sends RLC SDUs to the PDCP layer after reassembly. AMD PDUs that could not be reordered into RLC SDUs are discarded. If missing AMDs are detected, the receiver may request the retransmissions of these using STATUS PDUs messages. These messages contain an acknowledgement for each of the received RLC PDUs, which may be positive (ACK) or negative (NACK). In RLC AM, flow control is performed using two windows: the transmitting window and the receiving window. The transmitting window stores the packets that have been sent to the receiver and have not yet been acknowledged. The receiving window stores the received packets. Furthermore, the AM RLC entity implements the following configuration parameters:

- pollPDU and pollByte: pollPDU indicates how many PDUs can be sent by the transmitter before requesting a STATUS PDU. In the same way, pollByte indicates how many bytes can be sent by the transmitter before requesting a STATUS PDU. Each time the transmitter sends an AMD PDU to the receiver, the variables PDU_WITHOUT_POLL (the number of AMD PDUs sent since the last time a STATUS PDU was requested) and BYTE_WITHOUT_POLL (the number of bytes sent since the last time a STATUS PDU was requested) are updated, and it is checked whether these variables have achieved a value greater than or equal to the values of pollPDU and pollByte, respectively. If so, the transmitter will request the sending of a STATUS PDU, by setting the poll bit of the next AMD PDU to 1. Moreover, the transmitter will reset the variables PDU_WITHOUT_POLL and BYTE_WITHOUT_POLL.
- t-PollRetransmit: This timer determines the amount of time the transmitter must wait before requesting a STATUS PDU. When the transmitter sends an AMD PDU with the poll bit set to 1 (a STATUS PDU request), the t-PollRetransmit is started or restarted, in case it was active. If a STATUS PDU is received before the timer expires, the transmitter will stop and reset the timer. Otherwise, the transmitter sends a AMD PDU with the poll bit set to 1. This AMD PDU could be new or, in case the transmitting window is full, a retransmission of the last AMD PDU of any AMD PDU from which the ACK has not been received.
- maxRetxThreshold: This parameter indicates the maximum number of retransmissions allowed for an AMD PDU.
- t-StatusPohibit: This timer determines the minimum period of time that must elapse between the sending of two consecutive STATUS PDUs. When the receiver sends a STATUS PDU to the transmitter, the t-StatusProhibit is started. The receiver cannot send a new STATUS PDU to the transmitter until the timer expires.
- t-Reordering: This timer indicates the refresh rate of the state variables (set of metrics used to monitor the size and the limit of the transmitting and receiving windows), detecting lost AMD PDUs.

## 3. Transmission Control Protocol

TCP is a transport layer protocol that follows a client–server architecture. This protocol is connection-oriented. Thus, it is necessary to establish a connection between the client and the server before beginning the transmission of information. The connection will be released when the communication is finished. The TCP is characterized by providing a reliable and orderly delivery of packets.

To verify that the information is received correctly, the TCP makes use of ACKs. In this way, if the transmitter receives the ACK of a segment, it will know that it has been received correctly; otherwise, the segment will be retransmitted to the receiver.

**Figure 3:**
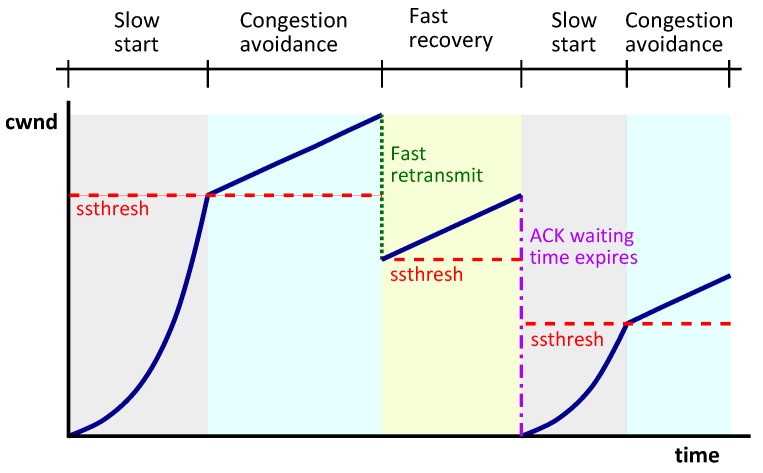
TCP congestion control algorithms.

In order to achieve high performance and to avoid network congestion [21], the TCP implements a transmission window and various congestion control algorithms. Moreover, the TCP uses two state variables for each connection: a congestion window (cwnd), limiting the amount of data that the transmitter can send before receiving an ACK, and the receiver’s advertised window (rwnd), indicating the free space available to store data in the receiver’s buffer. The minimum size between cwnd and rwnd limits the amount of data that can be transmitted through the network. Next, the TCP congestion control algorithms are described, as shown in Figure 3.

- Slow start: This algorithm consist of exponentially increasing the size of the cwnd, starting with a small size, determined by the maximum segment size (MSS) that can be transmitted by the sender, until a threshold called the slow start threshold (ssthresh), determined by the maximum size of the rwnd, is reached. If the network becomes congested during this phase, the value of the ssthresh will be updated as half of the minimum size between rwnd and cwnd, and the slow start algorithm will be restarted. This algorithm is used when the cwmd is smaller than the ssthresh.
- Congestion avoidance: Once the slow start phase is finished, the congestion avoidance phase is started. The congestion avoidance algorithm lineally increases the cwnd until the network becomes congested. In this phase, there are two reasons for network congestion: the reception of three duplicate ACKs or the expiration of the waiting time of receipt of an ACK. In the first case, the size of the cwnd and the ssthresh will be reduced and the linear increase will continue. In the second case, the size of the cwnd is reset and the slow start algorithm is started.
- Fast retransmit: If three duplicate ACKs are received, it is determined that the loss of a segment has occurred. When this occurs, the retransmission of the lost segment will be performed, without the need to wait for the retransmission timer to expire.
- Fast recovery: After the retransmission performed by the previous algorithm, the cwnd will continue its growth in the avoidance congestion phase.

## 4. Services

In this section, the main characteristics of the services used in this paper are presented. In the same way, the metrics used to evaluate the QoE perceived by users in each of these services are defined. The services considered are real-time video streaming and file transfer.

### 4.1. Real-Time Video Streaming Service

In a real-time video streaming service, the multimedia content is sent in a constant flow of data to the client as it is provided by the server. In this type of service, the content is reproduced as the data reach the client. For this reason, it is important that the data arrive on time, avoiding possible delays. Furthermore, in a real-time video streaming service, as the packets are played back, they are discarded; thus, the client does not get the complete multimedia file. This type of service is multicast oriented, similar to a television channel.

QoE assessment in video services is usually performed using the mean opinion score (MOS). In this method, users rate the video quality using the following values: 1 (bad), 2 (poor), 3 (fair), 4 (good), and 5 (excellent). The traditional way to calculate the MOS consists of testing a group of users in a controlled environment; this method of computing the MOS is known as subjective MOS. However, this method presents some issues, as it is slow and expensive. To solve these problems, empirical mathematical models have been developed. These models use network or service performance metrics (e.g., the peak signal to noise ratio (PSNR) and the structural similarity (SSIM)) in order to obtain an approximation of real users’ quality perception.

As in [17], in this work, the model defined in [22] to approximate the MOS is used. In addition to the PSNR, this model considers aspects such as the packet loss rate and the frame degradation during transmission. As indicated in [22], this method achieves a Pearson correlation of 0.9509 with the subjective MOS, obtaining more accurate results than traditional MOS estimation techniques based only on the use of PSNR or SSIM.

(1) $$MOS=4.367-0.5040\cdot \frac{d}{dPSNR}-0.0517\cdot l,$$

where *d* is the percentage of degraded frames, *dPSNR* is the PSNR average value of the distorted frames, and *l* is the lost frame rate.

In this paper, a frame is considered as degraded when its PSNR is below the fifth percentile of the video frames’ PSNR sent previously.

### 4.2. File Transfer Service

A file transfer service consists of the transmission of digital files through a communication channel from one system to another. The file transfer protocol (FTP) may be used to perform file transfers over TCP networks. FTP has a client–server architecture, where a client sends or receives files from the server. A file transfer service has strict requirements in terms of packet loss rates.

The most extended metrics used to evaluate the QoE perceived by users in a file transfer service are the download time, the end-to-end (E2E) throughput and the MOS. Although several models for the estimation of the subjective MOS for a file transfer service can be found in the literature, all of them have in common a dependence on the E2E throughput perceived by the users [23]. In this line, [24] shows that the perception of QoE of file transfer service clients presents a logarithmic relationship with the throughput achieved in transmission and with the file download time. In this work, with the aim of providing a broader view and not focusing on a specific MOS model for FTP services, the E2E throughput achieved by users has been used as an indicator of the QoE perceived by them.

## 5. QoE Optimization Algorithm

In order to automatize the optimization of the QoE based on the adjustment of RLC configuration parameters, a controller-based method has been selected. In particular, TM has been used. TM is one of the most widely used tools in the field of designing engineering experiments.

TM is an iterative method that calculates the value to be adopted by the input variables, which are called factors or parameters, depending on the value to be obtained for a given response metric. In this way, TM is based on using the response obtained in the previous iteration to readjust the values of the input parameters. The method ends when a certain stop criterion is achieved.

To solve the problem posed in this paper, RLC configuration parameters are used as TM factors (input variables). These configuration parameters vary depending on whether an AM or UM RLC mode of operation is used. The TM response metric—i.e., the metric to be optimized by the algorithm—is a measure of the QoE. As discussed in the previous section, this measure of the QoE is the MOS (see Section 4.1) in the case of the video service and the E2E throughput (see Section 4.2) in the case of the file transfer service. For each RLC configuration parameters, an initial range of values is established; i.e., maximum and minimum values that the parameter can adopt. In addition, the number of levels to be tested in each iteration for each parameter must be defined. This refers to the possible values that each input parameter can take in an iteration.

The levels of the parameters or factors used in each experiment are determined by an orthogonal array (OA) [25]. Thus, this matrix has as many columns as the number of input parameters of the optimization algorithm and as many rows as the number of experiments to be performed in each iteration of the algorithm. In each element of the matrix, the level that a given parameter may adopt in a specific experiment is stored. TM can be used with three objectives: (a) to minimize the response metric (the-smaller-the-better (STB)), (b) to achieve a target value of the response metric (the-nominal-the-best (NTB)), (c) to maximize the response metrics (the-larger-the-better (LTB)). This paper is focused on the LTB case, since the QoE is considered to be better the higher its value.

In order to obtain the optimal values of the studied RLC configuration parameters, TM uses a metric called the signal-to-noise ratio (SNR) [25]. This metric is computed internally by the TM algorithm after each iteration for each RLC configuration parameter and level. The optimal parameter level is the one that maximizes the SNR. The use of the SNR provides robustness to the algorithm, minimizing the effect of external factors [26]. This metric is calculated differently depending on the objective pursued (STB, NTB, or LTB). In the LTB case, the SNR metric is calculated as follows:

(2) $$SNR=10\cdot \log_{10}\left(y^{2}\right)$$

where *y* is the value of response metric (QoE) in each experiment.

Algorithm 1 shows the steps to follow during the QoE optimization process. The first step is the selection of the number of RLC configuration parameters, *N*, that will be adjusted and an OA, OArr, that fits the needs of the problem; i.e., an OA that sets the number of levels that are considered appropriate for each RLC configuration parameter, $numLevels=\{levels_{1},\ldots ,levels_{N}\}$. Then, a mapping between the levels of each RLC configuration parameter and the real values of these is made. To this end, it is necessary to define the maximum values, $maxValues=\{max_{1},\ldots ,max_{N}\}$, and minimum values, $minValues=\{min_{1},\ldots ,min_{N}\}$, to be tested for each parameter and to compute the step sizes between the levels, $stepSize=\{size_{1},\ldots ,size_{N}\}$ (the difference between two adjacent values of a parameter). Afterwards, the experiments indicated in the OA matrix are carried out obtaining a SNR value for each experiment (lines 5–8 of Algorithm 1). Subsequently, to calculate the SNR associated with each level of each RLC parameter, the SNR value obtained in each experiment in which that level was used is averaged (lines 9–19 of Algorithm 1). As a result, the combination of values of the RLC configuration parameters that provide the maximum result is obtained, $optimumValues=\{values_{1},\ldots ,values_{N}\}$. Next, the stop criterion, SC, is checked. The optimization process is finished if SC is satisfied. Otherwise, the search for the optimal values of the RLC configuration parameters continues. To do so, the following step is to narrow down the value ranges of each input parameter. For this, the optimal value of each parameter obtained in the previous iteration is taken as the central value, and the stepSize is reduced by multiplying it by the reduction factor RF∈(0,1)⊂R. From here, a new iteration would be performed starting with the mapping of the levels of the different RLC configuration parameter using their new ranges of values.
**Algorithm 1** Taguchi’s method for QoE optimization.1:**procedure**QoEoptimizationAlgorithm(*N*, OArr, $maxValues=\{max_{1},\ldots ,max_{N}\}$, $minValues=\{min_{1},\ldots ,min_{N}\}$, $numLevels=\{levels_{1},\ldots ,levels_{N}\}$, SC, RF)2:        $stepSize\leftarrow \frac{maxValues-minValues}{numLevels+1}$3:        **while** checkStopCriterion(SC, stepSize, *N*) **do**4:           Map OArr levels with RLC configuration parameters values5:           **for** row←1,len(OArr) **do**6:              Conduct experiment and obtain the resulting QoE, *y*7:              $SNR\leftarrow 10\cdot \log_{10}\left(y_{row}^{2}\right)$8:           **end for**9:           **for** n←1,N **do**10:              averageSNRList11:              **for** $level\leftarrow 1,numLevels_{n}$ **do**12:                    Compute the average SNR, snr13:                    averageSNRList.append(snr)14:              **end for**15:              optimumLevel←max(averageSNR)16:              Map the factor optimumLevel with its real value, val17:              $optimumValues_{n}\leftarrow val$18:              stepSize←stepSize·RF19:           **end for**20:        **end while**21:        **return** optimumValues22:**end procedure**
23:**procedure**checkStopCriterion(SC, stepSize, *N*)24:        meet←True25:        **for** i←1,N **do**26:           **if** $stepSize_{i}>SC$ **then**27:              meet←False28:           **end if**29:        **end for**30:        **return** meet31:**end procedure**


## 6. Proof of Concept

In this section, the results of the optimization process are shown. The optimization process for the two selected types of services (real-time video streaming and file transfer) was carried out in a simulated cellular environment. On the one hand, real-time video streaming services use the RLC UM mode, since it presents strict requirements in terms of latency. On the other hand, file transfer services use the RLC AM mode, since it is characterized by strict requirements in terms of the packet loss rate. Furthermore, to test the viability of the proposed optimization method in a real network, the optimization of the QoE perceived by the user of a file transfer service was performed using an LTE testbed, UMAHetNet (see Appendix A for more details).

### 6.1. Simulated Scenario

Firstly, the optimization of the QoE perceived by the user of a real-time video streaming service based on the adjustment of the RLC UM transmission buffer size was performed. Secondly, the optimization of the QoE perceived by the user of a file transfer service based on the adjustment of some of the RLC AM parameters was tested. The AM RLC parameters used were the t-PollRetransmit and the t-StatusProhibit timers. The selection of these parameters was made based on the performance of the RLC AM and following the study carried out in [17].

To perform the tests in the simulated environment the LTE module of the ns-3 simulator, called LENA [27], was used. LENA is an open-source LTE/evolved packet core (EPC) network simulator. The Evalvid tool [28] was used to simulate the real-time video streaming service and to evaluate its performance.

#### 6.1.1. Experiment Setup

The simulation scenario used to assess the optimization algorithm proposed in this paper was the same as that used in [17]. This scenario consisted of two LTE cells with a distance of 100 m between them. Each cell served 10 users randomly located in the coverage area of the cells. One of these users was used to evaluate the algorithm. In the first case, this user was the client of a real-time video streaming service. The streamed video was in MPEG-4 format, with a size of 3.6 MB, a duration of 10 s, and a rate of 30 fps. Packets with a delay of more than 150 ms were considered lost, thus complying with the requirements of real-time video transmission [29]. In the second case, the user was the client of a file transfer service. For these tests, 5 MB files were used. The rest of the users were in both cases clients of a constant bitrate service. Table 1 lists the main configuration parameters of the simulations.

#### 6.1.2. Results and Discussion

In the real-time video streaming service case, to configure the TM-based controller, the transmission buffer size of the RLC UM was selected as a factor or parameter to optimize and the MOS as the response metric to maximize. The initial range of values to be evaluated for the transmission buffer size ranged from 1 kB to 100 kB. For this configuration parameter, three levels were defined. The TM stop criterion was set to 0.5 as, with this value, a tradeoff between the optimization accuracy and the processing time was achieved. In the simulated scenario for very low system bandwidth values, the network would not have enough resources to meet the quality requirements of the real-time video streaming service in an acceptable way, and very low MOS values would be obtained at all times. Otherwise, when the bandwidth available in the system was very large in the simulated scenario, the network would be able to comfortably meet the quality requirements of the real-time video streaming service, minimizing the impact of the RLC layer’s configuration on QoE. For this reason, two intermediate bandwidth values were selected in order to observe the benefits of a proper adjustment of the transmission buffer size of the RLC UM on the QoE of the real-time video streaming service: 5 MHz and 10 MHz.

In an environment in which the packet generation rate is higher than the packet delivery rate, the QoE deterioration in a real-time video streaming service may be associated with the size of the RLC UM transmission buffer. On the one hand, if the RLC UM transmission buffer size were too small, a collapse of the buffer will be produced, causing the discarding of packets due to the lack of available space in the buffer. This would lead to an increment in the packet loss rate and, therefore, to a degradation in the QoE for the user. On the other hand, if the RLC UM transmission buffer size were too large, there would be an increase in the delay of sending packets due to the accumulation of packets in the buffer. In real-time services, packets that accumulate more than a certain delay are discarded. In this way, an excessive size of the RLC UM transmission buffer would also cause an increase in packet losses and, therefore, the deterioration of the QoE.

**Figure 4:**
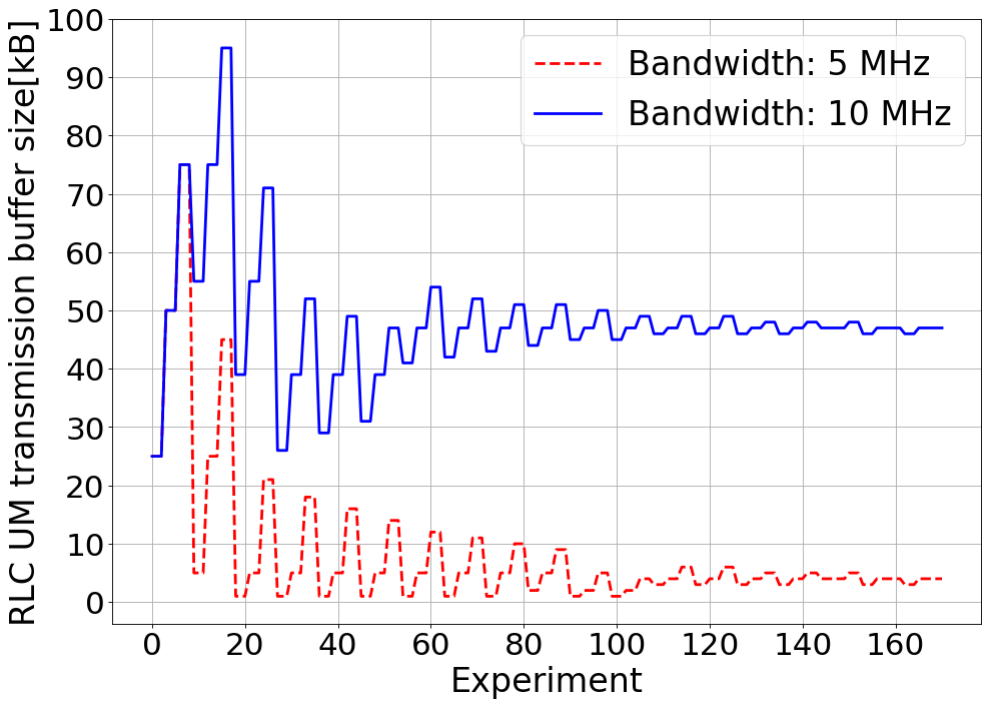
Simulated scenario—RLC UM transmission buffer adjustment.

In Figure 4, the buffer size used in each of the experiments performed during the optimization process is depicted. It can be observed that the optimization algorithm progressively reduces the search range for the transmitter buffer size values until it finds the value with which the user experiences the maximum QoE. With a 5 MHz system bandwidth, the optimal value for the transmitter buffer size is 4 kB, while with a 10 MHz system bandwidth, this value increases to 47 kB. The difference between the optimal buffer sizes for the different values of bandwidth available in the system is motivated by the fact that the more bandwidth is available in the system, the more data can be transmitted by the video server in each TTI. This means that in each TTI, more data of the RLC UM transmission buffer can be sent, and therefore, the increase of the delay produced by the accumulation of packets in the buffer will occur for a larger buffer size.

**Figure 5:**
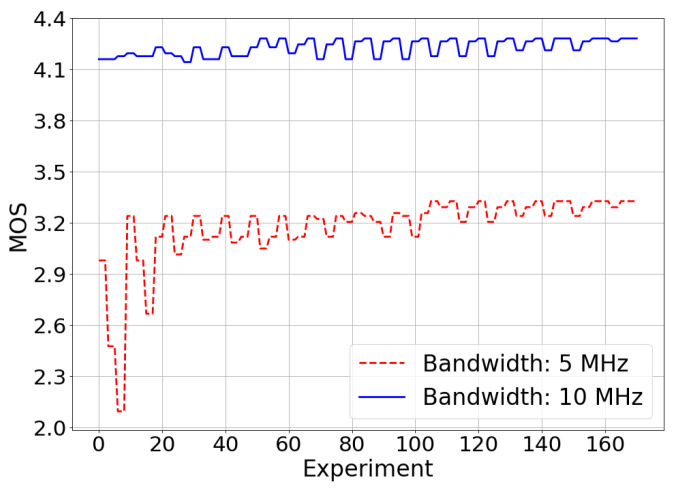
Simulated scenario—MOS optimization.

For a constant server delivery rate, the lower the bandwidth available in the system, the more losses will occur since the RLC UM transmission buffer will fill more quickly. As a result, the users will experience a worse QoE when the bandwidth available in the system is lower. This results in smaller MOS values when the available bandwidth in the system is 5 MHz and larger values when the available bandwidth in the system is 10 MHz. In Figure 5, it is shown that the MOS value obtained in the experiments performed during the optimization process converges to a maximum value. With 5 MHz system bandwidth, the maximum value reached by the MOS is 3.32, while with 10 MHz system bandwidth, it is 4.28. It is also possible to observe in Figure 5 that less MOS improvement is found for the 10 MHz use case with respect to the 5 MHz case. This is because, in the 10 MHz use case, the starting conditions are good (the network has more resources available).

In the file transfer service case, to configure the TM-based controller, the t-StatusProhibit and the t-PollRetransmit were selected as factors or parameters to optimize and the E2E throughput as the response metric to maximize. The initial range of values to be evaluated for t-StatusProhibit spanned from 0 ms to 600 ms, whereas the initial range of values to be evaluated for t-PollRetransmit spanned from 5 ms to 600 ms. The number of levels configured for each of these parameters was 3. The optimization process was terminated when the step size of all input parameters was less than or equal to 0.2. This value was calculated using heuristic methods, reaching a balance between model accuracy and processing time. As the file transfer service presented more relaxed quality requirements than the real-time video streaming service, in this case, the bandwidth values selected to show the benefits of adjusting the RLC AM configuration parameters over the QoE presented by the file transfer service clients were 1.4 MHz and 3 MHz.

QoE degradation in a file transfer service may be associated with a misconfiguration of RLC AM parameters. In RLC AM, control packets (STATUS PDUs) have priority in transmission over data packets (AMD PDUs). Likewise, retransmissions of AMD PDUs have priority over sending new AMS PDUs. The sending of an excessive number of STATUS PDUs and retransmissions can cause an increase in the delay of sending new information and, therefore, a deterioration in the QoE perceived by the user. As mentioned above, the t-PollRetransmit and t-StatusProhibit timers are used to control the frequency with which STATUS PDUs are sent. On the one hand, if these timers have very small values, the sending of STATUS PDUs will be very frequent, producing an increase in the delay of sending AMD PDUs. On the other hand, if the values of these timers are very large, STATUS PDUs will be sent infrequently, which can cause the transmission window to collapse and, therefore, lead to an increase in the packet loss rate.

**Figure 6:**
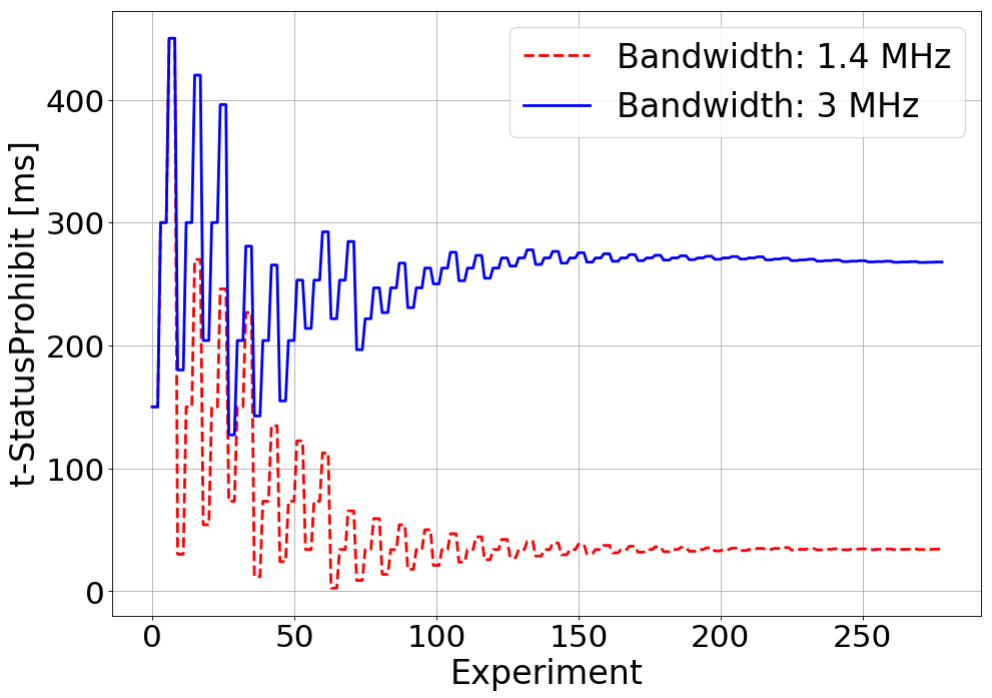
Simulated scenario—t-StatusProhibit adjustment.

**Figure 7:**
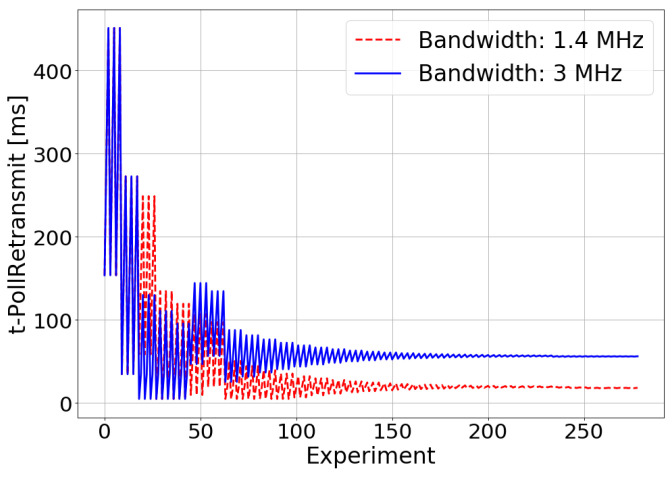
Simulated scenario—t-PollRetrasmit adjustment.

**Figure 8:**
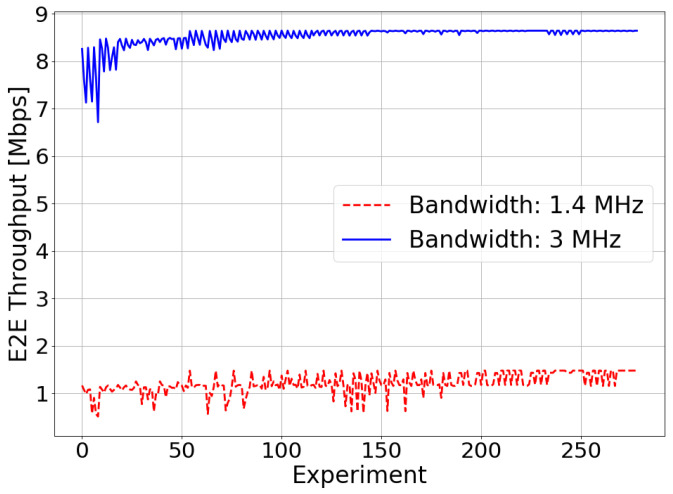
Simulated scenario—E2E throughput optimization.

Figure 6, Figure 7 and Figure 8 show the results of the experiments performed during the optimization process for bandwidths of 1.4 MHz and 3 MHz. From these figures, it can be inferred that the TM-based controller works correctly, providing the values of the RLC AM configuration parameters (t-StatusProhibit and t-PollRetransmit) that maximize the E2E throughput. These values are, in the 1.4 MHz case, 18 ms for t-PollRetransmit and 34.05 ms for t-StatusProhibit, and in the 3 MHz case, 56.12 ms and 267.52 ms. The difference between the optimal configuration parameters values for the different values of bandwidth available in the system is due to the effect of the RLC layer on the TCP layer operation. In the TCP, the loss or delays of the ACKs cause the throughput to fall. Due to the operation of the congestion control algorithm of the TCP, the throughput fall will be more abrupt when the bandwidth available in the system is greater. As shown above, in RLC AM, the sending of STATUS PDUs has priority over the sending of AMD PDUs. The ACKs of the TCP protocol are sent as AMD PDUs at the RLC level. Therefore, the delivery of STATUS PDUs will have priority over the delivery of TCP ACKs. In this way, the greater the bandwidth available in the system, the more sensitive it will be to the loss of throughput due to the excess of sending STATUS PDU. The smaller the values of the t-StatusProhibit and t-PollRetransmit timers, the greater the frequency of sending STATUS PDUs, which is why the optimal values of these timers are higher for a higher bandwidth.

The maximum E2E throughput achieved in the optimization process is 1.45 Mbps in the 1.4 MHz bandwidth case and 8.64 Mbps in the 3 MHz bandwidth case. The difference between these values is due to the amount of radio resources that it is possible to assign to the user in each scenario. When the bandwidth available in the system is higher, the user will have more radio resources to transmit and receive data, thus obtaining a higher E2E throughput.

### 6.2. Real Network

In order to evaluate the optimization algorithm in a real environment, some tests using the UMAHetNet network were carried out. In this case, only the optimization based on the adjustment of the RLC layer in AM mode was performed. As in the optimization of the QoE for the file transfer service in the simulated environment, the RLC AM parameters that were selected to carry out the optimization tasks were t-StatusProhibit and t-PollRetransmit.

#### 6.2.1. Experiment Setup

The service selected for the tests was a file transfer service. To avoid large values of delay and jitter in the results, the FTP server was installed on a computer with a public IP connected to the UMAHetNet network. To facilitate the analysis of the results, the FTP downloads were conducted with 20 MB files instead of 5 MB files as, in the selected scenario, for FTP downloads of 5 MB files, the transmission of the information was so fast that it did not allow the effects of the RLC layer to be observed efficiently. The considered scenario consisted of two LTE cells (a serving cell and an interfering cell), each one with one user. Only the user connected to the serving cell was analyzed. The user connected to the serving cell was a MONROE node [30], which is Linux-based user equipment (UE) that gathers performance indicators. The user connected to the interfering cell was a smartphone. The main configuration parameters of the UMAHetNet are summarized in Table 2.

#### 6.2.2. Results and Discussion

Like the tests carried out in the simulated environment, the parameters or factors selected to configure the TM-based controller were the RLC AM timers t-StatusProhibit and t-PollRetransmit. As the initial ranges of values to be evaluated for these two parameters, the ranges of values allowed in the network were selected, which in the case of t-StatusProhibit ranged from 0 ms to 500 ms and in the case of t-PollRetransmit spanned from 5 ms to 500 ms. For each configuration parameter, three levels were defined. The TM stop criterion was set to 0.2. The response metric to be maximized in this case was the E2E throughput obtained in file transfer experiments.

**Figure 9:**
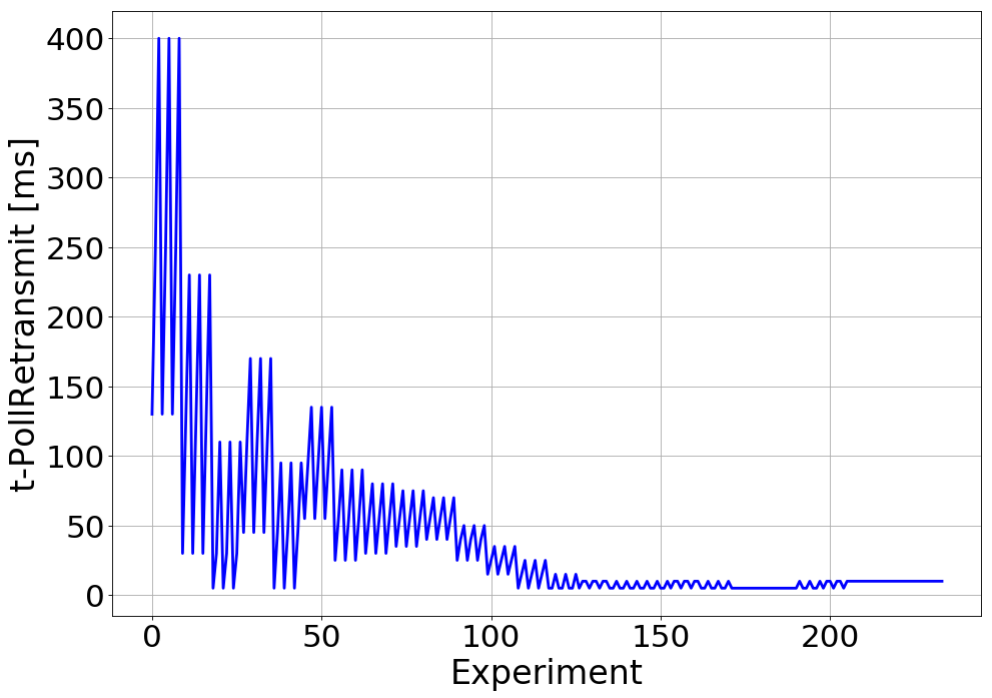
Real environment—t-PollRetrasmit adjustment.

**Figure 10:**
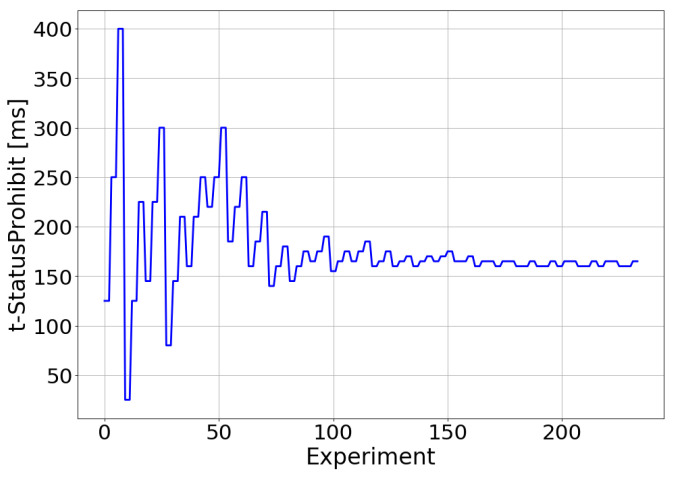
Real environment—t-StatusProhibit adjustment.

Figure 9 and Figure 10 show the adjustment of t-PollRetransmit and t-StatusProhibit, respectively. Thus, the values adopted for each of these parameters in the experiments performed during the optimization process are shown. In these figures, the functioning of the optimization algorithm can be observed. The TM-based controller progressively reduced the search range to find the combination of t-PollRetransmit and t-StatusProhibit that maximized the E2E throughput. For the tested scenario, this combination of parameters matched a t-PollRetransmit value of 9.53 ms and a t-StatusProhibit value of 162.4 ms.

**Figure 11:**
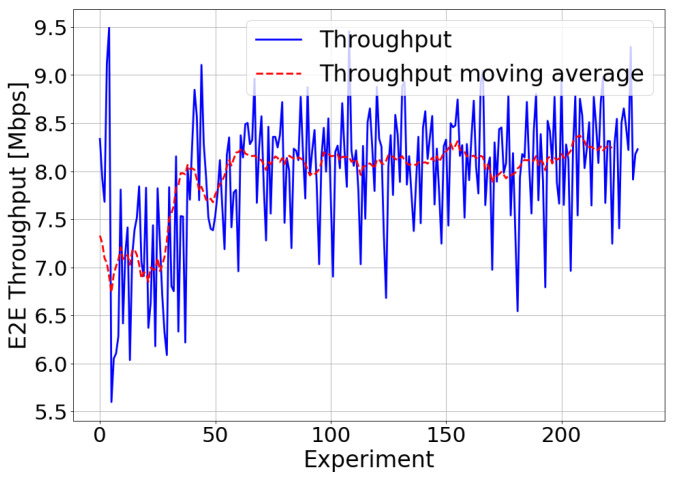
Real environment—E2E throughput optimization.

Finally, Figure 11 shows the E2E throughput obtained in each of the experiments performed during the optimization process (in blue) and its moving average (in red). It can be seen that the maximum throughput value was obtained for those values of the timers selected by the TM-based controller. As in the tests carried out in the simulator for the file transfer service, QoE optimization was achieved by reaching a tradeoff between excessively small and large values of the t-StatusProhibit and t-PollRetransmit timers. If the timers were set to very small values, there would be excessive control traffic, and the delay in sending the information would increase. Excessively large timer values would lead to the lack of acknowledgment of received packets and therefore the collapse of the transmission window and the loss of packets. The optimal values obtained in the t-StatusProhibit and t-PollRetransmit test are specific to this scenario and may vary depending on the environment conditions in which the algorithm is applied.

## 7. Conclusions

This paper presents a QoE optimization algorithm based on the adjustment of the RLC layer configuration. This algorithm has been evaluated using two types of services with different characteristics: on the one hand, a real-time video streaming service; on the other hand, a file transfer service. These two services usually run on different RLC layer operation modes. The optimization process of these two services has been carried out in a simulated environment. Moreover, the optimization of the QoE perceived by the user of a file transfer service has been performed in a real environment.

The RLC layer configuration, both in AM and UM, has a great impact on the losses and the latency experienced by the sent packets. A correct adjustment of the configuration parameters has been studied in these two RLC modes: the transmission buffer size in the RLC UM case and the t-StatusProhibit and t-PollRetransmit timers in the RLC AM case, providing a balance between the losses and the delays suffered by these packets and leading to an improvement in the QoE perceived by the users of the services analyzed.

The controller used to carry out the optimization process provides the values that should be adopted for the RLC configuration parameters to maximize the QoE perceived by the user. To this end, the controller only needs to know the configuration parameters’ initial search range of values. The optimization process has been performed for different values of bandwidth available in the system, thus proving that the optimal values of the configuration parameters and the maximum QoE experienced by the user are related to the scenario load conditions.

The tests carried out in the real network (UMAHetNet) have served to verify the correct functioning of the optimization algorithm in a commercial network, confirming the possibility of optimizing the QoE perceived by users of a file transfer service; in this case, by adjusting the RLC layer in AM mode.

In scenarios in which users make use of more than one service at the same time, if these services were carried by different data radio bearers (DRBs), the QoE optimization of the services should be performed separately. Thus, it would be necessary to select a different configuration for each RLC entity associated with a DRB. This selection of RLC parameters would be performed based on what has been learned by running the proposed optimization algorithm in different scenarios and using different services.

The algorithm presented in this paper can be used as an input to a decision support system for RLC layer configuration. One of the main lines of future work is the development of an algorithm that is capable of automatically adapting to changes in the scenario and dynamically modifying the RLC layer configuration.

## Figures and Tables

**Table 1:** Configuration of ns-3 scenario.

		Real-Time Video Streaming Service	File Transfer Service
Scenario		2 LTE cells 10 users in each cell Distance between eNBs 100 m	
System bandwidth		5 and 10 MHz	1.4 and 3 MHz
RLC mode		UM	AM
RLC parameters		Transmission buffer size: 1–100 kB	t-StatusProhibit: 0–600 ms t-PollRetransmit: 5–600 ms
Mobility		Static users	
Transmission direction		Download	
Services	Studied user	Real-time video streaming service (MPEG-4, 3.6 MB, 30 fps)	File transfer service (file 5 MB)
	Other users	Constant bitrate	Constant bitrate
e-NodeB		Omnidirectional antennas SISO, EIRP${}_{max}$= 30 dBm	
Scheduler		Proportional Fair	
Transmission Time Interval (TTI)		1 ms	
Maximum delay allowed in real-time video service		150 ms	-

**Table 2:** Configuration of the UMAHetNet network.

Scenario	2 Terminals (A MONROE Node and a Smartphone) Each Connected to a Picocell
System bandwidth	20 MHz
Serving cell reference signal transmission power	−7.6 dBm
Interfering cell reference signal transmission power	−14.9 dBm
RLC mode	AM
RLC parameters	t-StatusProhibit: 0–500 ms t-PollRetransmit: 5–500 ms
Mobility	Static user
Transmission direction	Download
Service	File transfer service (file size: 20 MB)
e-NodeB (Picocell)	Omnidirectional antennas MIMO
Scheduler	Enhanced proportional fair
TTI	1 ms

**Figure A1:**
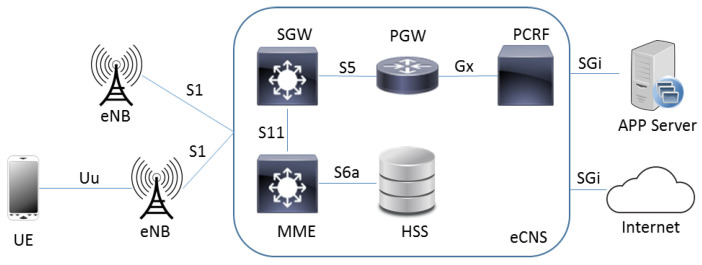
General scheme of the LTE network deployed at the University of Malaga.

## Abbreviations

The following abbreviations are used in this manuscript:

3GPP	Third Generation Partnership Project
5G	Fifth generation
AM	Acknowledged mode
AMD	Acknowledged mode data
E2E	End-to-end
eCNS	Evolved core network solution
EPC	Evolved packet core
FTP	File transfer protocol
HSS	Home subscriber server
KPI	Key performance indicator
KQI	Key quality indicator
LMT	Local management terminal
LTB	The-larger-the-better
LTE	Long term evolution
MME	Mobility management entity
MMS	Maximum segment size
MOS	Mean opinion score
NTB	The-nominal-the-best
OA	Orthogonal array
OAM	Operation, administration, and maintenance
P-GW	Packet data network gateway
PCRF	Policy and charging rules function
PDCP	Packet data convergence protocol
PDU	Protocol data units
PSNR	Peak signal to noise ratio
QoE	Quality of experience
RAN	Radio access network
RLC	Radio link control
RRC	Radio resource control
S-GW	Serving gateway
SDU	Service data unit
SNR	Signal-to-noise ratio
SON	Self-organizing networks
SSIM	Structural similarity
STB	The-smaller-the-better
TCP	Transmission control protocol
TM	Taguchi’s method
TTI	Transmission time interval
UDP	User data protocol
UE	User equipment
UM	Unacknowledged mode
UMD	Unacknowledged mode data
UMTS	Universal mobile telecommunications system

## Appendix A

UMAHetNet [31] is a full indoor LTE network. The RAN consists of 12 picocells (base stations). The EPC is composed of all core network elements: a home subscriber server (HSS), mobility management entity (MME), serving gateway (S-GW), packet data network gateway (P-GW), and a policy and charging rule function (PCRF) server. These elements are grouped into a single compact device, namely the evolved core network solution (eCNS), as shown in Figure A1. Moreover, UMAHetNet incorporates an operations, administration, and maintenance (OAM) system that allows the whole network to be managed.

The connection between all the network elements is performed by the use of a 24-port GB switch, allowing other servers to be added and providing all of the equipment access to the Internet.

Both the picocells and the eCNS are fully configurable. The picocells can be managed either directly through a local management terminal (LMT) client or by means of the U2000 and eCNS clients. The latter two clients allow the user to fully customize all the network parameters, as well as monitor the network status using built-in and user-defined KPIs. Furthermore, this testbed allows the user to integrate their own self-organizing network (SON) applications and algorithms, from self-configuration techniques to any self-optimizing and self-healing application.
